# Supplementary material for: Modeling glioblastoma heterogeneity as a dynamic network of cell states
Source: Mol Syst Biol. 2021 Sep 16;17(9):e10105. doi: 10.15252/msb.202010105 (PMC8444284; doi:10.15252/msb.202010105)
Supplement: Supplementary file 5 — Source Data for Figure 3 [file MSB-17-e10105-s001.zip › Figure3A_sourcedata/GSEA_3065/nosigdb_state2.GseaPreranked.1622718419215/gsea_report_for_na_neg_1622718419215.html]

Report for na\_neg 1622718419215 [GSEA]

| GS  follow link to MSigDB | GS DETAILS | SIZE | ES | NES | NOM p-val | FDR q-val | FWER p-val | RANK AT MAX | LEADING EDGE || 1 | LARSSON\_STATE\_2 | Details ... | 85 | -0.91 | -3.41 | 0.000 | 0.000 | 0.000 | 527 | tags=89%, list=6%, signal=94% |
| 2 | DOROSZKO\_U3180MG\_CLUSTER\_2 | Details ... | 465 | -0.64 | -2.97 | 0.000 | 0.000 | 0.000 | 1408 | tags=50%, list=15%, signal=56% |
| 3 | VANGRONINGEN\_MES | Details ... | 381 | -0.66 | -2.94 | 0.000 | 0.000 | 0.000 | 1348 | tags=48%, list=14%, signal=53% |
| 4 | OLSEN\_SCPLIKE | Details ... | 78 | -0.79 | -2.94 | 0.000 | 0.000 | 0.000 | 929 | tags=63%, list=10%, signal=69% |
| 5 | NB\_KI\_CLUSTER18 | Details ... | 70 | -0.79 | -2.89 | 0.000 | 0.000 | 0.000 | 869 | tags=63%, list=9%, signal=69% |
| 6 | LARSSON\_STATE\_3 | Details ... | 86 | -0.76 | -2.89 | 0.000 | 0.000 | 0.000 | 545 | tags=73%, list=6%, signal=77% |
| 7 | DOROSZKO\_U3054MG\_CLUSTER\_2 | Details ... | 234 | -0.63 | -2.72 | 0.000 | 0.000 | 0.000 | 1367 | tags=45%, list=14%, signal=52% |
| 8 | OLSEN\_MESENCHYMAL | Details ... | 69 | -0.75 | -2.67 | 0.000 | 0.000 | 0.000 | 806 | tags=58%, list=9%, signal=63% |
| 9 | NB\_KI\_CLUSTER17 | Details ... | 128 | -0.64 | -2.59 | 0.000 | 0.000 | 0.000 | 1371 | tags=48%, list=15%, signal=55% |
| 10 | RICHARDS\_VERHAAK\_CANCERCELL\_2010\_MESENCHYMAL | Details ... | 96 | -0.65 | -2.54 | 0.000 | 0.000 | 0.000 | 1821 | tags=55%, list=19%, signal=68% |
| 11 | RICHARDS\_NOWAKOWSKI\_SCIENCE\_2017\_ASTROCYTE\_UPREG | Details ... | 485 | -0.55 | -2.53 | 0.000 | 0.000 | 0.000 | 1263 | tags=37%, list=13%, signal=41% |
| 12 | IVY\_CTMVP | Details ... | 49 | -0.73 | -2.45 | 0.000 | 0.000 | 0.000 | 966 | tags=55%, list=10%, signal=61% |
| 13 | DOROSZKO\_PERIVASCULAR\_INVASION | Details ... | 36 | -0.76 | -2.43 | 0.000 | 0.000 | 0.000 | 674 | tags=50%, list=7%, signal=54% |
| 14 | ANASTASSIOU\_MULTICANCER\_INVASIVENESS\_PMID\_22208948 | Details ... | 28 | -0.78 | -2.39 | 0.000 | 0.000 | 0.000 | 1571 | tags=75%, list=17%, signal=90% |
| 15 | DOROSZKO\_U3013MG\_CLUSTER\_0 | Details ... | 28 | -0.78 | -2.39 | 0.000 | 0.000 | 0.000 | 674 | tags=57%, list=7%, signal=61% |
| 16 | GANDAL\_IBD\_UP\_PMID29439242 | Details ... | 55 | -0.68 | -2.38 | 0.000 | 0.000 | 0.000 | 1415 | tags=51%, list=15%, signal=60% |
| 17 | NB\_KI\_CLUSTER2 | Details ... | 254 | -0.55 | -2.37 | 0.000 | 0.000 | 0.000 | 2203 | tags=50%, list=23%, signal=63% |
| 18 | RICHARDS\_ZHONG\_NATURE\_2018\_ASTROCYTES\_UPREG | Details ... | 232 | -0.55 | -2.36 | 0.000 | 0.000 | 0.000 | 1452 | tags=43%, list=15%, signal=50% |
| 19 | NB\_KI\_CLUSTER23 | Details ... | 247 | -0.55 | -2.35 | 0.000 | 0.000 | 0.000 | 888 | tags=34%, list=9%, signal=37% |
| 20 | RICHARDS\_LIDDELOW\_NATURE\_2017\_A2\_REACTIVE\_ASTROCYTES | Details ... | 28 | -0.77 | -2.35 | 0.000 | 0.000 | 0.000 | 751 | tags=46%, list=8%, signal=50% |
| 21 | IVY\_CTPAN |  | 98 | -0.59 | -2.30 | 0.000 | 0.000 | 0.000 | 1323 | tags=39%, list=14%, signal=45% |
| 22 | RICHARDS\_NOWAKOWSKI\_SCIENCE\_2017\_OPC\_UPREG |  | 322 | -0.51 | -2.27 | 0.000 | 0.000 | 0.000 | 1461 | tags=39%, list=15%, signal=45% |
| 23 | LARSSON\_STATE\_5 |  | 99 | -0.59 | -2.27 | 0.000 | 0.000 | 0.000 | 605 | tags=45%, list=6%, signal=48% |
| 24 | SEGERMAN\_MULTITHERAPY\_RESISTANCE\_FIG5A\_PMID\_27974212 |  | 23 | -0.79 | -2.25 | 0.000 | 0.000 | 0.000 | 1296 | tags=70%, list=14%, signal=80% |
| 25 | RICHARDS\_NEFTEL\_CELL\_2019\_MES1 |  | 38 | -0.69 | -2.24 | 0.000 | 0.000 | 0.000 | 798 | tags=47%, list=8%, signal=52% |
| 26 | GANDAL\_AUTISM\_UP\_PMID29439242 |  | 80 | -0.61 | -2.24 | 0.000 | 0.000 | 0.000 | 1578 | tags=52%, list=17%, signal=63% |
| 27 | SUVA\_MES1\_PMID\_31327527 |  | 38 | -0.69 | -2.22 | 0.000 | 0.000 | 0.000 | 798 | tags=47%, list=8%, signal=52% |
| 28 | DOROSZKO\_U3054MG\_CLUSTER\_5 |  | 98 | -0.57 | -2.21 | 0.000 | 0.000 | 0.000 | 1880 | tags=48%, list=20%, signal=59% |
| 29 | RICHARDS\_ZHONG\_NATURE\_2018\_MICROGLIA\_UPREG |  | 198 | -0.53 | -2.20 | 0.000 | 0.000 | 0.000 | 1145 | tags=30%, list=12%, signal=34% |
| 30 | DEPRETER\_LOW\_NB\_RISK |  | 18 | -0.83 | -2.19 | 0.000 | 0.000 | 0.000 | 528 | tags=44%, list=6%, signal=47% |
| 31 | RICHARDS\_NOWAKOWSKI\_SCIENCE\_2017\_NEN\_EARLY2\_UPREG |  | 394 | -0.48 | -2.19 | 0.000 | 0.000 | 0.000 | 2132 | tags=41%, list=23%, signal=50% |
| 32 | WANG\_MESENCHYMAL\_PMID\_28697342 |  | 24 | -0.75 | -2.19 | 0.000 | 0.000 | 0.000 | 1935 | tags=79%, list=20%, signal=99% |
| 33 | DALMO\_EXTINCTION\_UNPUB |  | 24 | -0.75 | -2.19 | 0.000 | 0.000 | 0.000 | 1935 | tags=79%, list=20%, signal=99% |
| 34 | RICHARDS\_NEFTEL\_CELL\_2019\_AC |  | 35 | -0.70 | -2.17 | 0.000 | 0.000 | 0.000 | 948 | tags=54%, list=10%, signal=60% |
| 35 | DOROSZKO\_U3180MG\_CLUSTER\_5 |  | 30 | -0.71 | -2.17 | 0.000 | 0.000 | 0.001 | 1083 | tags=47%, list=11%, signal=53% |
| 36 | SUVA\_AC\_PMID\_31327527 |  | 35 | -0.70 | -2.17 | 0.000 | 0.000 | 0.001 | 948 | tags=54%, list=10%, signal=60% |
| 37 | ALLEN\_111\_ASTROL1\_6FGFR3ETNPPL |  | 24 | -0.74 | -2.16 | 0.000 | 0.000 | 0.001 | 1135 | tags=63%, list=12%, signal=71% |
| 38 | RICHARDS\_NOWAKOWSKI\_SCIENCE\_2017\_VRG\_UPREG |  | 382 | -0.46 | -2.10 | 0.000 | 0.000 | 0.001 | 1461 | tags=36%, list=15%, signal=41% |
| 39 | DOROSZKO\_U3013MG\_CLUSTER\_3 |  | 100 | -0.55 | -2.10 | 0.000 | 0.000 | 0.001 | 1410 | tags=46%, list=15%, signal=54% |
| 40 | LARSSON\_STATE\_1 |  | 99 | -0.54 | -2.09 | 0.000 | 0.000 | 0.001 | 806 | tags=36%, list=9%, signal=39% |
| 41 | RICHARDS\_ZHONG\_NATURE\_2018\_OPC\_UPREG |  | 74 | -0.57 | -2.08 | 0.000 | 0.000 | 0.001 | 1024 | tags=42%, list=11%, signal=47% |
| 42 | ALLEN\_116\_OLIGOL4\_6OPALIN |  | 22 | -0.74 | -2.07 | 0.000 | 0.000 | 0.001 | 1840 | tags=68%, list=19%, signal=84% |
| 43 | ALLEN\_113\_ASTROL1FGFR3FOS |  | 24 | -0.72 | -2.04 | 0.000 | 0.000 | 0.001 | 1083 | tags=71%, list=11%, signal=80% |
| 44 | DOROSZKO\_U3054MG\_CLUSTER\_1 |  | 17 | -0.76 | -2.02 | 0.000 | 0.000 | 0.002 | 960 | tags=53%, list=10%, signal=59% |
| 45 | ALLEN\_112\_ASTROL1FGFR3MT1G |  | 25 | -0.68 | -2.01 | 0.000 | 0.000 | 0.002 | 1135 | tags=68%, list=12%, signal=77% |
| 46 | DOROSZKO\_U3013MG\_CLUSTER\_1 |  | 36 | -0.62 | -1.99 | 0.000 | 0.000 | 0.003 | 1375 | tags=44%, list=15%, signal=52% |
| 47 | RICHARDS\_NOWAKOWSKI\_SCIENCE\_2017\_IPC\_NEN1\_UPREG |  | 231 | -0.46 | -1.98 | 0.000 | 0.000 | 0.003 | 1615 | tags=40%, list=17%, signal=47% |
| 48 | ALLEN\_114\_OPCL1\_6MYT1 |  | 16 | -0.76 | -1.97 | 0.000 | 0.000 | 0.006 | 1127 | tags=75%, list=12%, signal=85% |
| 49 | NB\_KI\_CLUSTER13 |  | 119 | -0.50 | -1.97 | 0.000 | 0.000 | 0.007 | 1819 | tags=39%, list=19%, signal=48% |
| 50 | RICHARDS\_NOWAKOWSKI\_SCIENCE\_2017\_MGE\_RG1\_UPREG |  | 320 | -0.44 | -1.94 | 0.000 | 0.000 | 0.008 | 1684 | tags=39%, list=18%, signal=46% |
| 51 | NB\_KI\_CLUSTER14 |  | 215 | -0.45 | -1.94 | 0.000 | 0.000 | 0.008 | 1886 | tags=39%, list=20%, signal=48% |
| 52 | RICHARDS\_LIDDELOW\_NATURE\_2017\_A1\_REACTIVE\_ASTROCYTES |  | 21 | -0.68 | -1.92 | 0.000 | 0.001 | 0.012 | 880 | tags=43%, list=9%, signal=47% |
| 53 | RICHARDS\_VERHAAK\_CANCERCELL\_2010\_CLASSICAL |  | 115 | -0.47 | -1.89 | 0.000 | 0.001 | 0.019 | 1769 | tags=43%, list=19%, signal=53% |
| 54 | TIISIG\_PMID\_32649888 |  | 25 | -0.63 | -1.87 | 0.003 | 0.001 | 0.025 | 1306 | tags=48%, list=14%, signal=56% |
| 55 | OUTERRADIALGLIA |  | 48 | -0.55 | -1.83 | 0.000 | 0.002 | 0.044 | 498 | tags=35%, list=5%, signal=37% |
| 56 | SUVA\_OPC\_PMID\_31327527 |  | 37 | -0.57 | -1.83 | 0.003 | 0.002 | 0.044 | 1354 | tags=54%, list=14%, signal=63% |
| 57 | SUVA\_MES2\_PMID\_31327527 |  | 44 | -0.56 | -1.82 | 0.000 | 0.002 | 0.045 | 1323 | tags=36%, list=14%, signal=42% |
| 58 | POLLEN\_OUTERRADIALGLIA\_PMID\_26406371 |  | 48 | -0.55 | -1.82 | 0.000 | 0.002 | 0.046 | 498 | tags=35%, list=5%, signal=37% |
| 59 | RICHARDS\_NEFTEL\_CELL\_2019\_MES2 |  | 44 | -0.56 | -1.81 | 0.000 | 0.002 | 0.046 | 1323 | tags=36%, list=14%, signal=42% |
| 60 | RICHARDS\_NEFTEL\_CELL\_2019\_OPC |  | 37 | -0.57 | -1.81 | 0.000 | 0.003 | 0.050 | 1354 | tags=54%, list=14%, signal=63% |
| 61 | LARSSON\_STATE\_6 |  | 101 | -0.46 | -1.80 | 0.000 | 0.003 | 0.054 | 923 | tags=27%, list=10%, signal=29% |
| 62 | DOROSZKO\_U3054MG\_CLUSTER\_0 |  | 60 | -0.51 | -1.78 | 0.000 | 0.003 | 0.066 | 1426 | tags=43%, list=15%, signal=51% |
| 63 | GAROFANO\_IAVARONE\_NEU\_SUBTYPE |  | 162 | -0.42 | -1.77 | 0.000 | 0.004 | 0.079 | 2335 | tags=42%, list=25%, signal=55% |
| 64 | SINGH\_SOX2\_DOWNREGULATED\_TRANSCRIPTS\_PMID\_28122245 |  | 90 | -0.47 | -1.76 | 0.000 | 0.004 | 0.086 | 955 | tags=29%, list=10%, signal=32% |
| 65 | NB\_KI\_CLUSTER15 |  | 156 | -0.43 | -1.76 | 0.000 | 0.004 | 0.089 | 2678 | tags=43%, list=28%, signal=59% |
| 66 | IVY\_IT |  | 32 | -0.56 | -1.75 | 0.003 | 0.005 | 0.104 | 561 | tags=22%, list=6%, signal=23% |
| 67 | DOROSZKO\_U3054MG\_CLUSTER\_3 |  | 195 | -0.42 | -1.74 | 0.000 | 0.005 | 0.109 | 1770 | tags=31%, list=19%, signal=38% |
| 68 | GANDAL\_BIPOLAR\_UP\_PMID29439242 |  | 63 | -0.48 | -1.74 | 0.003 | 0.005 | 0.115 | 1437 | tags=48%, list=15%, signal=56% |
| 69 | PARADA\_T1\_DN\_26461091 |  | 40 | -0.54 | -1.74 | 0.003 | 0.005 | 0.115 | 1846 | tags=45%, list=20%, signal=56% |
| 70 | ALMSTEDT\_ALK |  | 51 | -0.51 | -1.74 | 0.000 | 0.005 | 0.117 | 961 | tags=25%, list=10%, signal=28% |
| 71 | RICHARDS\_NOWAKOWSKI\_SCIENCE\_2017\_EN\_PFC3\_UPREG |  | 200 | -0.41 | -1.73 | 0.000 | 0.005 | 0.126 | 1606 | tags=36%, list=17%, signal=42% |
| 72 | ANGIOGENESIS\_GO\_0001525 |  | 20 | -0.62 | -1.73 | 0.008 | 0.005 | 0.129 | 1303 | tags=40%, list=14%, signal=46% |
| 73 | NB\_KI\_CLUSTER20 |  | 17 | -0.63 | -1.71 | 0.010 | 0.006 | 0.150 | 2503 | tags=59%, list=27%, signal=80% |
| 74 | ALLEN\_115\_OLIGOL4\_6MOBPCOL18A1 |  | 24 | -0.59 | -1.70 | 0.013 | 0.007 | 0.155 | 1163 | tags=54%, list=12%, signal=62% |
| 75 | NB\_KI\_CLUSTER11 |  | 50 | -0.49 | -1.70 | 0.003 | 0.007 | 0.168 | 1744 | tags=44%, list=18%, signal=54% |
| 76 | GANDAL\_SCHIZOPHRENIA\_UP\_PMID29439242 |  | 79 | -0.46 | -1.69 | 0.000 | 0.007 | 0.179 | 1434 | tags=48%, list=15%, signal=56% |
| 77 | DOROSZKO\_U3054MG\_CLUSTER\_7 |  | 28 | -0.56 | -1.65 | 0.014 | 0.011 | 0.259 | 2065 | tags=46%, list=22%, signal=59% |
| 78 | RICHARDS\_CAHOY\_JNEUROSCI\_2011\_ASTRO\_MATURE |  | 497 | -0.36 | -1.65 | 0.000 | 0.011 | 0.260 | 1856 | tags=28%, list=20%, signal=33% |
| 79 | WANG\_CLASSICAL\_PMID\_28697342 |  | 37 | -0.51 | -1.64 | 0.017 | 0.011 | 0.274 | 1452 | tags=35%, list=15%, signal=41% |
| 80 | DOROSZKO\_U3054MG\_CLUSTER\_10 |  | 244 | -0.37 | -1.62 | 0.000 | 0.013 | 0.312 | 1614 | tags=28%, list=17%, signal=33% |
| 81 | DOROSZKO\_U3180MG\_CLUSTER\_6 |  | 38 | -0.49 | -1.60 | 0.017 | 0.014 | 0.351 | 1003 | tags=29%, list=11%, signal=32% |
| 82 | RICHARDS\_NOWAKOWSKI\_SCIENCE\_2017\_IN\_CTX\_CGE2\_UPREG |  | 225 | -0.37 | -1.58 | 0.000 | 0.016 | 0.389 | 1407 | tags=29%, list=15%, signal=33% |
| 83 | GANDAL\_ALCOHOLADDICTION\_DN\_PMID29439242 |  | 59 | -0.43 | -1.55 | 0.010 | 0.021 | 0.477 | 1211 | tags=32%, list=13%, signal=37% |
| 84 | RICHARDS\_NOWAKOWSKI\_SCIENCE\_2017\_NIN1\_UPREG |  | 149 | -0.37 | -1.54 | 0.000 | 0.021 | 0.487 | 807 | tags=21%, list=9%, signal=22% |
| 85 | NB\_KI\_CLUSTER8 |  | 313 | -0.35 | -1.54 | 0.000 | 0.022 | 0.503 | 2095 | tags=38%, list=22%, signal=47% |
| 86 | DOROSZKO\_U3180MG\_CLUSTER\_1 |  | 17 | -0.58 | -1.53 | 0.048 | 0.023 | 0.523 | 1126 | tags=41%, list=12%, signal=47% |
| 87 | GANDAL\_BIPOLAR\_DN\_PMID29439242 |  | 19 | -0.56 | -1.52 | 0.033 | 0.024 | 0.537 | 1530 | tags=37%, list=16%, signal=44% |
| 88 | IVY\_LE |  | 24 | -0.51 | -1.52 | 0.033 | 0.025 | 0.550 | 511 | tags=17%, list=5%, signal=18% |
| 89 | RICHARDS\_NOWAKOWSKI\_SCIENCE\_2017\_NIN3\_UPREG |  | 140 | -0.36 | -1.48 | 0.000 | 0.032 | 0.642 | 1462 | tags=28%, list=15%, signal=32% |
| 90 | KRONA\_ROSEN\_WHITE\_MATTER\_INVASION |  | 194 | -0.33 | -1.37 | 0.006 | 0.067 | 0.896 | 870 | tags=19%, list=9%, signal=21% |
| 91 | RICHARDS\_NOWAKOWSKI\_SCIENCE\_2017\_IN\_STR\_UPREG |  | 176 | -0.33 | -1.36 | 0.007 | 0.068 | 0.906 | 1581 | tags=27%, list=17%, signal=32% |
| 92 | KRONA\_ROSEN\_PERIVASCULAR\_INVASION |  | 47 | -0.41 | -1.34 | 0.070 | 0.077 | 0.935 | 770 | tags=17%, list=8%, signal=18% |
| 93 | GANDAL\_ALCOHOLADDICTION\_UP\_PMID29439242 |  | 139 | -0.32 | -1.31 | 0.026 | 0.092 | 0.963 | 1147 | tags=19%, list=12%, signal=21% |
| 94 | PARADA\_T2\_DN\_26461091 |  | 39 | -0.39 | -1.27 | 0.132 | 0.117 | 0.986 | 2200 | tags=44%, list=23%, signal=57% |
| 95 | NB\_KI\_CLUSTER6 |  | 479 | -0.27 | -1.25 | 0.014 | 0.129 | 0.988 | 2299 | tags=32%, list=24%, signal=40% |
| 96 | RICHARDS\_NEFTEL\_CELL\_2019\_NPC1 |  | 36 | -0.39 | -1.25 | 0.158 | 0.129 | 0.991 | 1116 | tags=33%, list=12%, signal=38% |
| 97 | RICHARDS\_NOWAKOWSKI\_SCIENCE\_2017\_IN\_CTX\_MGE2\_UPREG |  | 197 | -0.30 | -1.24 | 0.027 | 0.133 | 0.993 | 1056 | tags=22%, list=11%, signal=24% |
| 98 | SUVA\_NPC1\_PMID\_31327527 |  | 36 | -0.39 | -1.24 | 0.142 | 0.137 | 0.994 | 1116 | tags=33%, list=12%, signal=38% |
| 99 | KLING\_GBM\_PHENO\_PMID\_27667176 |  | 143 | -0.31 | -1.23 | 0.069 | 0.138 | 0.996 | 1383 | tags=31%, list=15%, signal=36% |
| 100 | TISIG\_PMID\_32649888 |  | 34 | -0.38 | -1.18 | 0.210 | 0.186 | 0.999 | 1378 | tags=32%, list=15%, signal=38% |
| 101 | RICHARDS\_NOWAKOWSKI\_SCIENCE\_2017\_NEN\_LATE\_UPREG |  | 356 | -0.26 | -1.16 | 0.063 | 0.210 | 1.000 | 1945 | tags=29%, list=21%, signal=35% |
| 102 | RICHARDS\_NOWAKOWSKI\_SCIENCE\_2017\_NIN5\_UPREG |  | 259 | -0.25 | -1.09 | 0.193 | 0.303 | 1.000 | 1294 | tags=22%, list=14%, signal=24% |
| 103 | RICHARDS\_NEFTEL\_CELL\_2019\_NPC2 |  | 32 | -0.34 | -1.06 | 0.374 | 0.352 | 1.000 | 1891 | tags=38%, list=20%, signal=47% |
| 104 | SUVA\_NPC2\_PMID\_31327527 |  | 32 | -0.34 | -1.04 | 0.346 | 0.378 | 1.000 | 1891 | tags=38%, list=20%, signal=47% |
| 105 | RICHARDS\_NOWAKOWSKI\_SCIENCE\_2017\_IPC\_NEN1\_UPREG\_1 |  | 136 | -0.26 | -1.03 | 0.316 | 0.395 | 1.000 | 1086 | tags=22%, list=12%, signal=25% |
| 106 | NB\_KI\_CLUSTER16 |  | 75 | -0.26 | -0.98 | 0.520 | 0.515 | 1.000 | 1572 | tags=27%, list=17%, signal=32% |
| 107 | GAROFANO\_IAVARONE\_MTC\_SUBTYPE |  | 225 | -0.23 | -0.98 | 0.536 | 0.519 | 1.000 | 1353 | tags=16%, list=14%, signal=18% |
| 108 | NB\_KI\_CLUSTER22 |  | 142 | -0.23 | -0.96 | 0.574 | 0.560 | 1.000 | 2833 | tags=40%, list=30%, signal=56% |
| 109 | GANDAL\_IBD\_DN\_PMID29439242 |  | 25 | -0.32 | -0.93 | 0.587 | 0.625 | 1.000 | 1083 | tags=28%, list=11%, signal=32% |
| 110 | NB\_KI\_CLUSTER12 |  | 41 | -0.23 | -0.74 | 0.903 | 0.933 | 1.000 | 138 | tags=5%, list=1%, signal=5% |
| 111 | NB\_KI\_CLUSTER24 |  | 191 | -0.16 | -0.67 | 1.000 | 0.966 | 1.000 | 2751 | tags=29%, list=29%, signal=40% |
Table: Gene sets enriched in phenotype **na **[plain text format]****
